# Peer tutoring during clinical internships as experienced by physiotherapy students: a meta-synthesis of qualitative studies

**DOI:** 10.1007/s10459-025-10488-7

**Published:** 2025-11-07

**Authors:** Simone Salvitti, Isabel Colado Gimeno, Alvisa Palese, Irene Mansutti, Mauro Di Bari

**Affiliations:** 1 Unit of Cardiorespiratory Physiotherapy, Azienda Sanitaria Universitaria Friuli Centrale (ASUFC), Udine, Italy; 2 Unit of Pediatric Physiotherapy, Azienda Sanitaria Universitaria Friuli Centrale (ASUFC), Udine, Italy; 3 Department of Medicine, University of Udine, Udine, Italy; 4 Department of Experimental and Clinical Medicine, University of Florence, Florence, Italy; 5 Unit of Geriatrics, Department of Medicine and Geriatrics, Azienda Ospedaliero-Universitaria Careggi, Florence, Italy

**Keywords:** Peer tutoring, Peer mentoring, Peer learning, Physiotherapy, Systematic review

## Abstract

Clinical internship is a core component of physiotherapy education. Peer tutoring, defined as a relationship where a more experienced student supports a less experienced one, encourages collaborative and mutual learning. However, no summary of qualitative studies has been provided to date regarding peer tutoring as experienced by physiotherapy students. A qualitative meta-synthesis was conducted. The study protocol was registered in PROSPERO (CRD420251004431), and the PRISMA guidelines were followed. Searches were conducted across four search engines, and included studies were evaluated using the Critical Appraisal Skills Programme (CASP) checklist. Data were analyzed through inductive thematic analysis, and the GRADE-CERQual tool was used to assess confidence in the findings. Out of 686 identified articles, four were included in the final analysis. Two central themes emerged: for mentors, “Actively participating in patient care,” and for mentees, “Developing a path of personal and professional growth.” The integrative synthesis also identified four additional themes: (A) Indirect influence of the clinical tutor on peer relationship dynamics; (B) Presence of barriers to peer collaboration; (C) Promotion of personal growth; and (D) Reduction of difficulties associated with acting alone. Clinical internships that include peer tutoring have both positive and negative effects, either alleviating or intensifying the challenges physiotherapy students encounter in complex healthcare settings. A well-structured peer tutoring program can benefit both mentors and mentees by enhancing learning and skills development. However, the dual role of clinical tutors as supervisors and evaluators may strain peer relationships and increase anxiety and stress among students. PROSPERO CRD420251004431 (5th March 2025).

**Supplementary Information:**

The online version contains supplementary material available at https://doi.org/10.1007/s10459-025-10488-7.

## Background

Undergraduate programs for future physiotherapists are provided through theoretical education and clinical internships, the latter being essential for developing students’ clinical competence and professional identity within a community of practice (Hafferty et al., 2015; Laitinen-Väänänen et al., 2007). In Italy, the healthcare sector responsible for the clinical training of future physiotherapists is under considerable strain. University programs and student numbers are increasing in response to workforce shortages (Zambelli, 2024), amid a progressive challenge in public healthcare sustainability (GIMBE, 2023). From 2012 to 2024, the number of physiotherapy students in Italy rose from 6,660 to 8,401, while the number of public healthcare-employed physiotherapists increased only marginally, from 17,083 to 18,500. Considering the required internship hours (1,500 per student) and the mandated 1:2 tutor-student ratio, approximately 1,400 clinical tutors are needed—an increase of about 26% compared to 2012 (Supplementary Information – Figure S1 and Figure S2). This trend risks creating a systemic overload, as the growing number of students exacerbates challenges in delivering adequate clinical experiences. Clinical tutors have been reported to experience significant stress related to student supervision (e.g., Bearman et al., 2013), while students experience heightened anxiety (Alzayyat & Al-Gamal, 2014) and inadequate internships learning opportunities (Stubbs & Soundy, 2013). Limited resources often make it difficult for clinical tutors to balance patient care with student supervision, which requires ongoing training, detailed feedback, and support (Jokelainen et al., 2011). In many cases, tutors may lack specialization in the clinical areas they supervise or formal training in tutorial methods; these factors may further threaten the quality of clinical training delivery (Hafferty et al., 2015). Overall, these challenges suggest the urgent need to redesign educational models for sustainability in both process and resources.

Peer education provided by senior students have been reported to play an important role in supporting both junior students and clinical tutors (Prothero et al., 2025). Peer tutoring (or mentoring, hereinafter tutoring) is defined as “a relationship in which a more experienced person helps a less experienced one to achieve desired outcomes” where individuals with different experiences and skills collaborate to generate mutual learning (Hunt & Ellison, 2010). The learning of junior students (or mentees) can be understood as the result of the complex dynamics of social interaction (support, dialogue, relationships, emotional engagement) and collaboration with more experienced senior peers (or mentors), who in turn are offered the opportunity to build confidence and cultivate an interest in clinical tutoring (Christiansen & Bell, 2010). Peer tutoring is considered one of the most influential factors in student learning within the clinical environment (Campbell et al., 1994; Carey et al., 2016) providing benefits (Sevenhuysen et al., 2017), including enhanced collaboration and emotional resilience even in complex work environments (Price & Witheside, 2016). Moreover, peer tutoring has been considered as facilitating co-regulated learning: socially shared regulation of learning is a metacognitive and adaptive mental process that is negotiated and fine-tuned in collaboration (Panadero & Järvelä, 2015; Hadwin et al., 2017).

Each change in the internship models, should consider the experience of students as the centre of the whole educational process valuing their perspective and involving them in co-redesign. Specifically, understanding physiotherapy students experience of learning activities may provide novel insight into how best to enhance their education from the perspective of the learner (Leahy et al., 2020). Moreover, their feedback may guide the development of innovative and sustainable clinical education models informing faculty decisions regarding how to improve the quality of educational experiences, and addressing challenges posed by evolving healthcare systems and limited resources. However, to our knowledge, no summary of qualitative studies has been provided to date regarding peer tutoring as experienced by physiotherapy students, while a systematic review is available for nursing students (Jacobsen et al., 2022). Therefore, this study was intended at filling in the gap to offer actionable insights to optimize the educational process and align it with the realities of healthcare delivery. The aim was to identify, analyze, and synthesize qualitative findings regarding the experiences of physiotherapy students participating in a peer tutoring program during their clinical internships. Specifically, the study addresses the following research questions:

1. How did junior students (mentees) experience supervision by fellow students during clinical internships, according to studies published to date?
2. How did senior students (mentors) experience their role when acting as mentors to their peers during clinical internships, according to studies published to date?

## Methods

A meta-synthesis based on systematic review was conducted and here reported according to the Cochrane Qualitative Evidence Synthesis guidelines (Glenton et al., 2023). The various phases of the meta-synthesis were designed and conducted in line with the recommendations by Sandelowski and Barroso (2018) and Ghirotto (2020). Moreover, the research protocol was registered in the International Prospective Register of Systematic Reviews (PROSPERO: CRD420251004431, 5th March 2025).

### Research team

A multidisciplinary research team was established, which included two physiotherapists (SS, ICG) and three senior researchers (AP, IM, MDB). All the investigators had longstanding academic experience in teaching and tutoring students of physiotherapy or other allied healthcare sciences. The senior researchers also had consolidated research expertise in primary and secondary qualitative research studies.

### Knowledge gap confirmation

In February 2025, a systematic search was conducted to identify the knowledge gap in “peer tutoring” during clinical internship experiences, firstly verifying that no existing studies summarized the qualitative evidence produced on this topic. A search string was specifically designed to query search engines and databases relevant to the study topic (Supplementary Information – Figure S3). No reviews synthesizing evidence on the experiences of physiotherapy students, either as mentees or mentors, during peer tutoring in clinical internships were found.

### Search string and databases

The SPIDER approach (Sample, Phenomenon of Interest, Design, Evaluation, Research type), specifically tailored for qualitative and mixed-methods research, was used to frame the research question for the database searches as an alternative to the PICO (Population/Patient/Problem, Intervention/Exposure, Comparison/Control, and Outcome) framework (Cooke et al., 2012). The search targeted qualitative studies exploring the experiences and perceptions of physiotherapy students in peer mentoring during clinical internships.

The search string used (Supplementary Information – Figure S4) was adapted to each search engine/database’s formatting criteria without altering its logical structure. Literature searches were conducted using four search engines: PubMed, Embase, Elton Bryson Stephens Company (EBSCO), and ProQuest. The databases queried included: Medline, Embase, Cumulative Index to Nursing and Allied Health Literature (CINAHL), Education Source Ultimate, APA (American Psychological Association) PsycInfo, Psychology and Behavioral Sciences Collection, Child Development and Adolescent Studies, Business Source Complete, Library and Information Science Source, Sociology Source Ultimate, Publicly Available Content Database, SciTech Premium Collection, Science Database, Coronavirus Research Database, and Psychology Database. The bibliographies of included studies were also reviewed, while gray literature was excluded.

The systematic search was performed on March 15, 2025, with no temporal publication restrictions, considering all available literature at the time. All articles were exported and managed in Microsoft Excel, where duplicates were removed.

### Eligibility criteria

Overall, studies were required to report clear and explicit research aims aligned with the review questions, as well as a clear presentation of data using themes and subthemes or categories and subcategories. The specific inclusion and exclusion criteria of the studies are summarized in Table 1.

**Table 1:** Inclusion and exclusion criteria

**Inclusion Criteria**: there were included
- Primary studies using qualitative or mixed methods designs (e.g., ethnography, phenomenology, case studies, grounded theory, qualitative process evaluations). - Employing qualitative data collection methods (focus groups, individual interviews, observations, diaries, document analysis, open-ended survey questions). - Applying qualitative data analysis methods (thematic analysis, framework analysis, grounded theory). - Reporting, with verbatim quotations, the experiences of (a) physiotherapy students involved in peer tutoring during clinical internships and/or (b) students mentored by more experienced peers.
**Exclusion criteria**: there were excluded studies
- Focused on clinical tutors or course instructors in peer mentoring. - Where peer tutor was limited to classroom-based activities (e.g., simulated exercises). - Published in non-peer-reviewed journals. - Collecting qualitative data analysed using descriptive statistics. - Unavailable in their full text. - Published in languages other than English due to lack of resources for accurate contextual translation, which would have required expert professionals such as cultural mediators.

After duplicate removal, two blinded reviewers (SS and ICG) independently assessed titles and abstracts against the inclusion and exclusion criteria. Further screening considered factors such as study design, objectives, and relevance of findings. Studies on clinical internship models with different tutor-student ratios (e.g., 2:1, 3:1, 4:1) were excluded unless they explicitly involved peer tutoring. If studies included perspectives beyond physiotherapy students (e.g., tutors or students from other disciplines), only data concerning physiotherapy students were considered.

### Quality assessment of included studies

Two blinded reviewers (SS and ICG) independently assessed methodological quality using the Critical Appraisal Skills Programme (CASP) checklist for qualitative research, which includes 10 questions evaluating study method, credibility, and relevance. Studies were categorized as “Very Low,” “Low,” “Moderate,” or “High” quality (Horntvedt et al., 2018). For mixed-methods studies, only the qualitative research components were assessed. Discrepancies were discussed with a third reviewer (AP) when necessary. To ensure an inclusive approach, studies were not excluded based on their methodological limitations Garside, 2014); instead, these limitations informed confidence in the review’s findings.

The Grading of Recommendations Assessment, Development and Evaluation - Confidence in the Evidence from Reviews of Qualitative research (GRADE-CERQual) approach evaluated confidence in synthesis findings based on methodological limitations, consistency, adequacy, and relevance (Lewin et al., 2018). Two reviewers (SS, ICG) independently assessed the findings, while a third author (AP) was consulted in case of discrepancies.

### Data extraction and analysis

Two blinded reviewers (SS and ICG) independently extracted data into a tabular format, including authorship, publication year, country, objectives, research questions, study design, data collection/analysis methods, participants, inclusion/exclusion criteria, clinical settings, and findings. Quotations were extracted in full and arranged alphabetically in an Excel support. When the process of data extraction has been completed, the thematic analysis (Byrne, 2022) guided qualitative analysis, following Braun and Clarke’s six-phase framework (Braun & Clarke, 2021) as following: (1) familiarization with data; (2) generating initial codes; (3) searching for themes; (4) reviewing and refining themes; (5) defining and naming themes and (6) reporting findings. An inductive approach was used by two researchers (SS, ICG), working independently and then together, letting themes emerge directly from the data. A recursive, iterative approach allowed movement between phases, ensuring flexibility and openness to emergent interpretations (Braun & Clarke, 2021).

For interpreting the data used in the synthesis process, the approach described by Malpass and colleagues (2009) was adopted. A secondary thematic analysis was conducted: quotations extracted from the different studies were considered to inductively generate subthemes and themes. Their frequency across studies was then calculated. Descriptions were provided for each. The meta-synthesis involved a high level of iteration, with a continuous “back-and-forth movement” between the participants’ quotations, the meanings emerging from the studies, and the reviewers’ conceptualizations (Barnett-Page & Thomas, 2009). Given that only one study (Paparella-Pitzel et al., 2021) clearly distinguished between mentee and mentor an iterative and interpretative process was applied to synthesize the qualitative findings of this study separately. Subsequently, data from multiple studies were incorporated to preserve the specificity of this study in relation to our review aims.

### Rigor, research collaboration and reflexivity

The systematic process was ensured by following the Preferred Reporting Items for Systematic Reviews and Meta-Analyses (PRISMA) guidelines (Page et al., 2021). Reviewers conducted all steps independently, followed by meetings to resolve disagreements. Continuous comparison of the findings was ensured through study retrieval, meaning the search for codes or other keywords within the quotations, allowing for re-reading considering the progression of the research and the acquisition of new information and insights (Salvini, 2015). It was not necessary to exclude any reviewer from the assessment of methodological limitations, as no studies authored by members of the research team were included. Reflexivity was valued throughout the entire data analysis process, both individually by keeping a diary of notes and as a multidisciplinary group during meetings. Discussions were also encouraged to share how reviewers’ experiences and beliefs influenced their interpretations (Edge, 2011).

## Findings

**Fig. 1:**
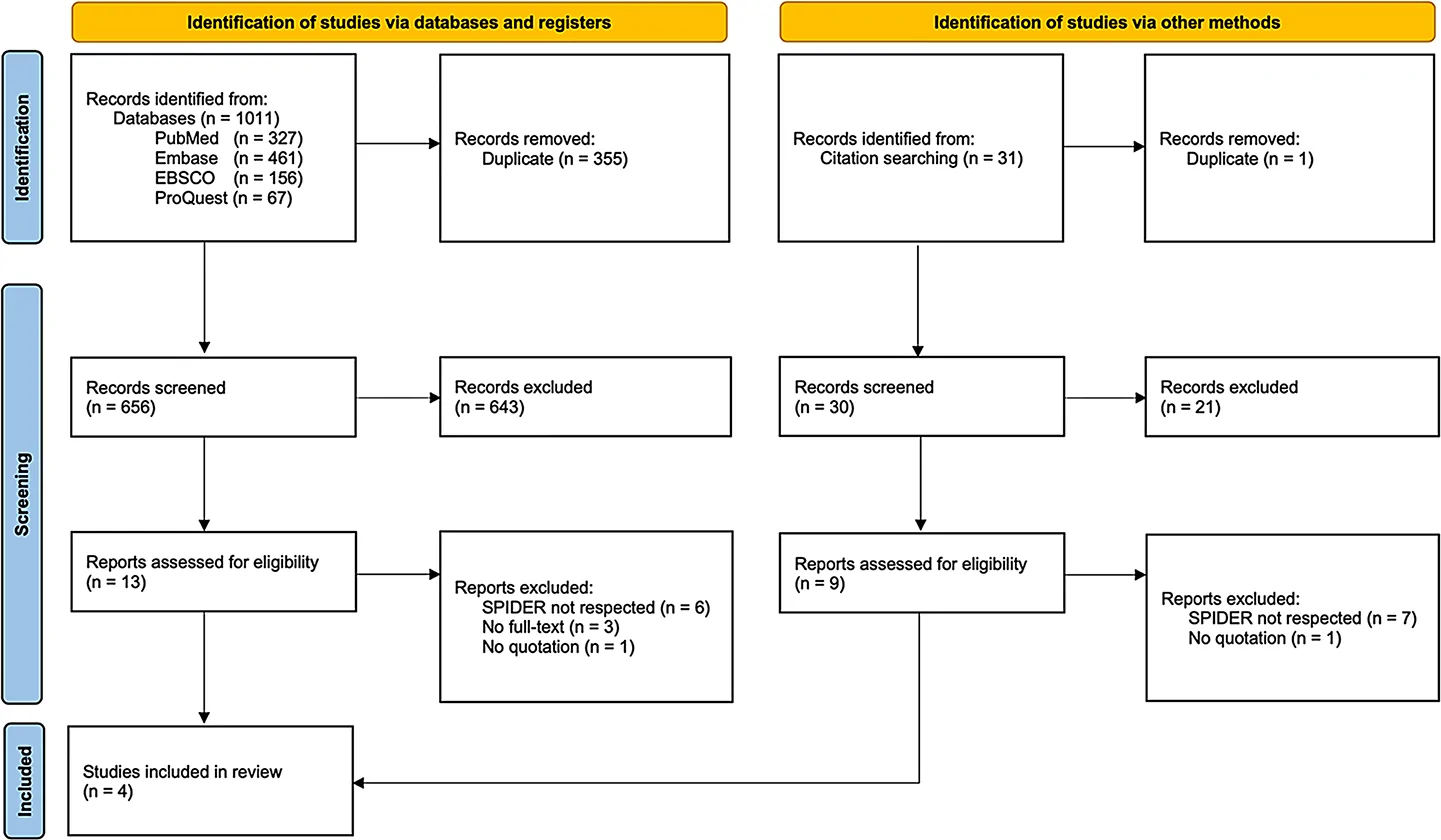
Preferred reporting items for systematic reviews and meta-analyses (PRISMA) flow diagram. Legend: EBSCO: Elton Bryson Stephens Company; SPIDER: Sample, Phenomenon of Interest, Design, Evaluation, Research type

The database searches yielded 656 non-duplicate and potentially relevant articles, with an additional 30 retrieved from other sources (cross-referencing), resulting in a total of 686 articles (Fig. 1). After screening titles and abstracts, 22 articles were selected for full-text reading. After exclusion of 18 articles, four studies were included (Sevenhuysen et al., 2015; Alpine et al., 2018; Paparella-Pitzel et al., 2021; Tailor et al., 2024).

### Study main characteristics and their quality

Studies published in English between 2015 and 2024 (Table 2) involved 8 to 110 students, all enrolled in physiotherapy degree programs at various stages of their academic education, in Europe, US and Australia. Two studies (Sevenhuysen et al., 2015; Tailor et al., 2024) used a qualitative research design, while two others (Alpine et al., 2018; Paparella-Pitzel et al., 2021) employed a mixed-methods approach. Data collection methods included semi-structured interviews (Tailor et al., 2024), focus groups (Sevenhuysen et al., 2015), and questionnaires with open-ended questions (Alpine et al., 2018; Paparella-Pitzel et al., 2021). One study (Sevenhuysen et al., 2015) compared students’ experiences in two different clinical placement settings: an informal peer learning model with a 2:1 student-to-clinical tutor ratio already in use, and a specifically structured PAL (Peer-Assisted Learning) model (Sevenhuysen et al., 2013). The other studies reported students’ experiences within variously defined “peer learning” contexts.

Regarding the research questions, only one study (Paparella-Pitzel et al., 2021) clearly distinguished between mentee and mentor, although both were considered peers as they shared the same student status. One study (Sevenhuysen et al., 2015) defined participants as peers because they were in the same academic year, without distinguishing between mentee and mentor roles. The remaining two studies, although referring to “peer learning” in their titles, did not clearly report the nature of student relationships or their roles in the reciprocal learning context during clinical placements (Tailor et al., 2024; Alpine et al., 2018), preventing a clear distinction between mentor, mentee, or peer as in the other two studies. Additionally, two studies (Paparella-Pitzel et al., 2021; Tailor et al., 2024) focused exclusively on physiotherapy students, while the other two included both students and clinical tutors (Sevenhuysen et al., 2015; Alpine et al., 2018).

The results of the evaluation using the CASP tool are reported in the Table S1 and Table S2 (Supplementary Information). Most studies met the expected quality in study aims, appropriateness of methodology, and research design. However, shortcomings emerged in recruitment strategies, data analysis, ethical considerations, and in defining the relationship between researchers and participants. Only two studies provided sufficient information about the researchers’ roles, occupations, and prior experience with qualitative research: one was conducted by experienced researchers in both data collection and analysis (Sevenhuysen et al., 2015), while the other was conducted by physiotherapy students (Tailor et al., 2024), resulting in a study design labeled by the authors as “Peer-to-Peer Qualitative Research.”

**Table 2:** Summary of the main characteristics of the included studies

Authors (year) Country	Aim	Research question	Design	Participants	Inclusion and exclusion criteria	Data collection	Data analysis	Clinical Setting	Summary of the findings
Sevenhuysen et al. (2015) Australia	To bring out the positive aspects of the PAL model with the goal of improving it	What are the experiences of students and clinical educators in a paired student placement model incorporating facilitated peer-assisted learning activities, compared to a traditional paired teaching approach?	Qualitative research	Sampling: Purposive [not explained] Sample: 22 students (12 F; 10 M) Age: 18–20 year (15), 20–25 year (7) Mentee/Mentor: Peer (same year)	Inclusion: The third-year students were studying for a 4-year undergraduate physiotherapy degree Exclusion: NR	Focus group	Thematic Analysis	Three acute hospitals, one sub-acute inpatient center and one outpatient rehabilitation center with internship in cardiorespiratory and neurology clinical placements	Students and educators each reported positive aspects of peer-assisted learning (such as reduced educator burden, greater productivity, and fostering of professional skills), although there were aspects of educator-facilitated learning that it could not replace
Alpine et al. (2018) Ireland	To investigate student and practice educator evaluations of practice placements using the 2 to 1 supervision framework and implementation model	NR	Mixed method: Cross-sectional pilot study using a self-reported questionnaire	Sampling: Purposive [not explained] Sample: 10 students (9 F; 1 M) from third (8) and final year (2) Age: NR Mentee/Mentor: NR	Inclusion: All students completing a 2 to 1 placement between the years 2013 and 2015 were eligible to participate in the pilot study Exclusion: NR	Open-ended questions trough an anonymized questionnaire	Content analysis	All placements were of 6-week duration in both inpatient and outpatient clinical settings. Placements were in a variety of clinical specialities including musculoskeletal, pediatrics, and neurology	There was generally positive agreement that placements using the 2 to 1 model were positively evaluated by participants
Paparella-Pitzel et al. (2021) USA, New Jersey	To report on the overall perceptions and experiences of students who participated in an SRPBC and on the discovered association and value of peer-to‐peer learning across all domains.	NR	Cross-sectional, mixed‐method survey study	Sampling: Purposive [not explained] Sample: 110 students (76 F; 34 M) from first (30,9%), second (23,6%) and third year (45,5%) of DPT programme Age: NR Mentee/Mentor: 1st year (mentee) and 2nd year students (mentor)	Inclusion: students in the 3rd, 2nd, and 1st year of the DPT program Exclusion: NR	Write-in responses from the open-ended questions of a questionnaire distributed via Qualtrics	Directed content analysis trough inductive and deductive ways with NVIVO 12 software	SRPBC	A total of 108 (27.8%) write-in responses were coded as comments about peer-to‐peer learning
Tailor et al. (2024) UK, England	To explore physiotherapy students’ perspectives of peer relationships during placements	NR	Peer-to-Peer Qualitative Research	Sampling: Purposive [not explained] Sample: 8 students Age: NR Mentee/Mentor: NR	Inclusion: consenting third year BSc Physiotherapy students who had participated in a multi-model placement Exclusion: Students who had shared a placement site, but had not worked together, or who had failed a multi-model placement	Online semi-structured interview via Microsoft Teams	Analytical approach (IPA)	NR	Multi-model placements provide social and emotional support to students, increasing their confidence Peer relationships present opportunities for collaborative working and academic support if they are adequately framed as such by the practice educator and wider team Students may benefit from university-based support to prepare them to maximize the peer relationship

NR: not reported; F: female; M: male; DPT: Doctor of Physical Therapy; IPA: Interpretive Phenomenology Analysis; PAL: Peer-Assisted Learning; SRPBC: Student-Run Pro Bono Clinic; USA: United States of America; UK: United Kingdom

### Meta-summary

A total of 44 quotations from physiotherapy students were extracted; four were excluded due to lack of relevance to the aim or research questions of this study, resulting in 40 quotations used for analysis (Supplementary Information - Table S3). Specifically, from Sevenhuysen et al. (2015), 10 quotations were extracted (8 included, two excluded); from Alpine et al. (2018), six (none excluded); from Paparella-Pitzel et al. (2021), 18 (16 included, two excluded); and from Tailor et al. (2024), 10 (none excluded). The data analysis identified four themes and 13 subthemes (Supplementary Information - Table S4) as follows.

#### Reduced difficulties in acting alone (4/4; 100%)

This theme captures how peer tutoring helps address the challenges students often face when learning independently in clinical settings. Having a peer present provides emotional reassurance, practical collaboration, and a more reflective, less intimidating learning environment. Three key subthemes emerged: peer tutoring as (1.1) Opportunity for peer discussion, (1.2) Mutual support, and (1.3) A safe environment. First, learning alongside a peer enabled joint clinical reasoning, shared decision-making, and collaborative reflection on practice. Students experienced deeper learning through exchanging ideas, co-developing care plans, and comparing and evaluating their own performance with that of their peers. Beyond academic collaboration, peers offered important emotional support. Relying on each other throughout the placement eased the transition into the clinical environment, promoted professional growth, and fostered strong interpersonal relationships. Additionally, peer collaboration created a psychologically safer environment than traditional student-supervisor dynamics. Students felt more comfortable expressing uncertainty, asking questions, and discussing clinical reasoning openly, which helped reduce stress and build confidence (Supplementary Information - Table S4).

#### Personal growth (4/4; 100%)

Peer-tutoring supported students’ professional and interpersonal development. Two main subthemes of growth emerged: (2.1) Enhancing feedback-giving skills and (1.2) Developing collaborative abilities. Students reported improved ability and confidence in giving and receiving feedback. Routinely exchanging feedback with a peer fostered openness, critical thinking, and reflection. Engaging in peer feedback allowed students to recognize areas for improvement in themselves and others, while learning to communicate constructively and respectfully. Learning alongside peers enabled students to strengthen their collaborative skills through shared decision-making, co-treatment, and joint problem-solving. Learning to navigate differences in clinical approaches, communicate effectively, and build professional relationships was seen as a valuable part of the peer-assisted experience. These collaborative experiences contributed not only to improved clinical reasoning but also to stronger interpersonal bonds and teamwork (Supplementary Information - Table S4).

#### Collaboration difficulties (4/4; 100%)

While peer-tutoring often positive interactions, students also identified several challenges in collaborative settings. These challenges primarily involved competitive dynamics, mismatched working styles, and situations where the peer relationship felt unproductive or imbalanced. Three main subthemes emerged: (3.1) Inappropriate competitiveness; (3.2) Differences in learning and working styles and (3.3) Perceived lack of usefulness. Some students experienced unhelpful competition within peer pairs, including attempts to outshine each other, unequal access to learning opportunities, and tensions when one peer dominated clinical tasks. The presence of another student sometimes triggered comparison or anxiety about performance and assessment, potentially undermining individual growth. Working closely with someone who had a different approach to learning or patient care was occasionally difficult to manage. These differences sometimes led to poor coordination, frustration, or inefficiencies in clinical practice, especially when students struggled to adapt or compromise. Some students, particularly those in junior roles, felt underutilized or excluded, which limited their engagement and learning. When responsibilities were not shared equally or appropriately tailored to each student’s level, the experience could feel redundant or discouraging (Supplementary Information - Table S4).

#### Relationship with the clinical tutor (3/4; 75%)

Students’ interactions with clinical tutors significantly influenced their learning experiences during placements. Two key subthemes emerged: (4.1) Fear of asking questions perceived as silly, and (4.2) Subjective or biased feedback. Students often hesitate to ask questions they considered basic or “silly” in front of clinical tutors due to concerns about being judged or negatively assessed. This fear acted as a barrier to learning and led students to seek clarification from peers instead, creating a safer space for uncertainty. Providing or receiving feedback—either from peers or involving peers—was sometimes complicated by personal relationships. Students felt uncomfortable raising concerns or giving constructive criticism when working closely with peers they considered friends. This dynamic occasionally hindered open communication and honest feedback, especially when issues needed to be escalated to supervisors (Supplementary Information - Table S4).

### Meta-synthesis

An integrative synthesis was conducted, focusing specifically on mentees’ and mentors’ perspectives and interpreting data beyond what was explicitly reported in each individual study (Supplementary Information, Table S5).

**Fig. 2:**
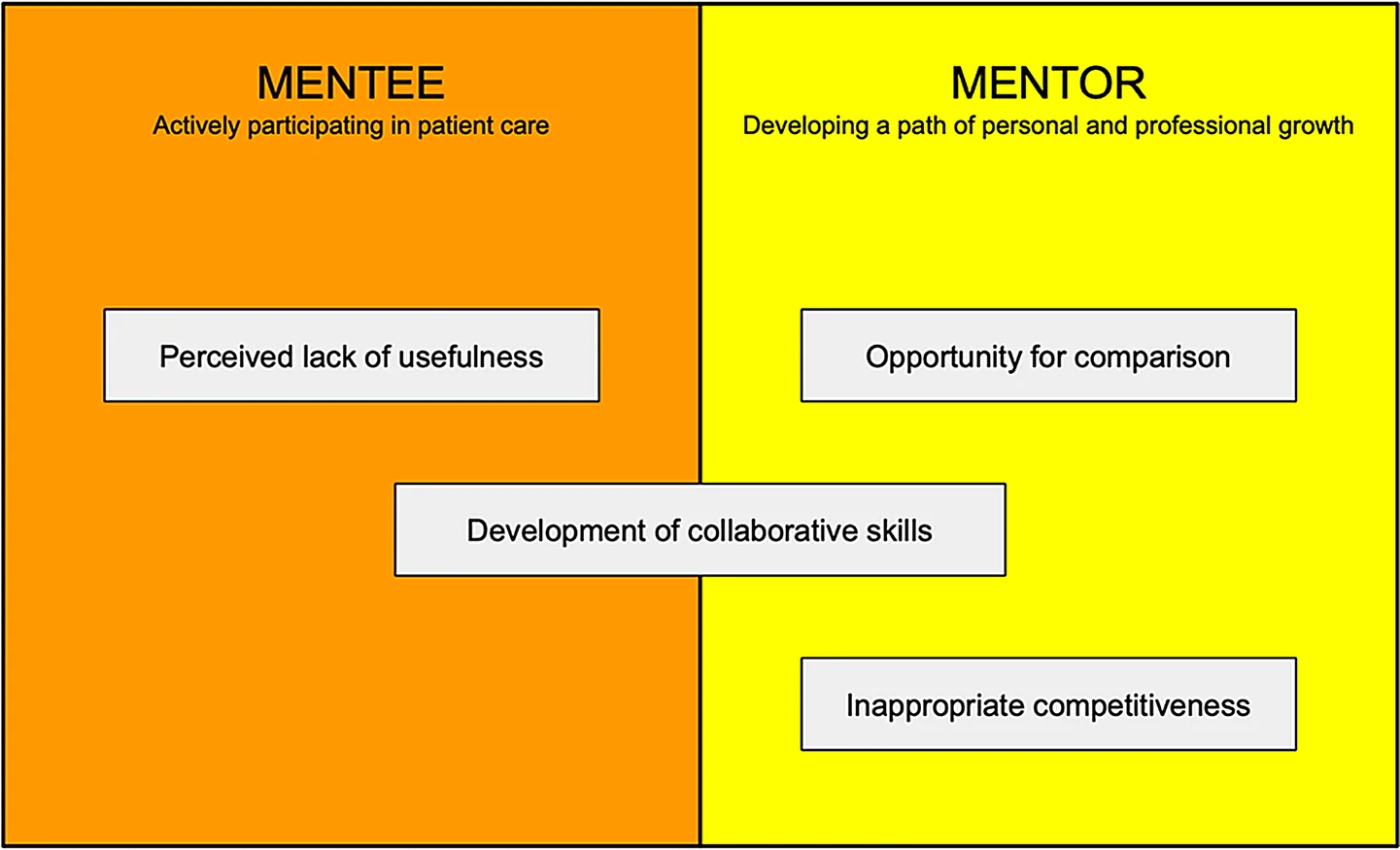
Themes and subthemes (grey boxes) emerged from the analysis of physiotherapy students’ experience during clinical peer internship experiences regarding mentor (yellow quadrant) and mentee (orange quadrant) (Paparella-Pitzel et al., 2021)

First, the study by Paparella-Pitzel et al. (2021) distinguished quotes from mentees and mentors. Analysis of these quotes revealed two main themes: for mentors, “Actively participating in patient care,” and for mentees, “Developing a path of personal and professional growth.” Each theme included different subthemes and one common subtheme (Fig. 2).

Mentees described experiences reflecting a desire to actively participate in patient care by collaborating with their mentors, whom they viewed as role models. One subtheme, “Perceived lack of usefulness,” indicated that mentees often wanted greater involvement in clinical practice rather than being limited to observation. When restricted to a passive role, they found the learning experience less meaningful or beneficial, emphasizing the importance of hands-on participation for their professional growth.

Mentors, in contrast, appeared more focused on personal and professional growth, achieved through interaction and collaboration with mentees in a mutual learning environment free from harmful competition among students. Two subthemes emerged: (1) “Opportunity for comparison”—mentors described collaboration with mentees as a valuable opportunity to reflect on and refine their own techniques. Observing mentees’ approaches allowed mentors to re-evaluate their methods, fostering professional self-awareness and continuous improvement; and (2) “Inappropriate competitiveness”—some mentors noted that mentees might sometimes attempt to take over or challenge the mentor’s role, introducing competition into the relationship. If not managed constructively, this dynamic could undermine the intended learning environment and the mentor’s guiding role.

A transversal theme, “Development of collaborative skills,” also emerged. Both mentors and mentees highlighted collaboration as a key element of the learning process. For mentees, working with mentors fostered a sense of partnership and accountability for patient outcomes. For mentors, engaging with mentees enhanced their ability to communicate, guide, and adapt their approach, strengthening the reciprocal nature of the mentoring relationship.

**Fig. 3:**
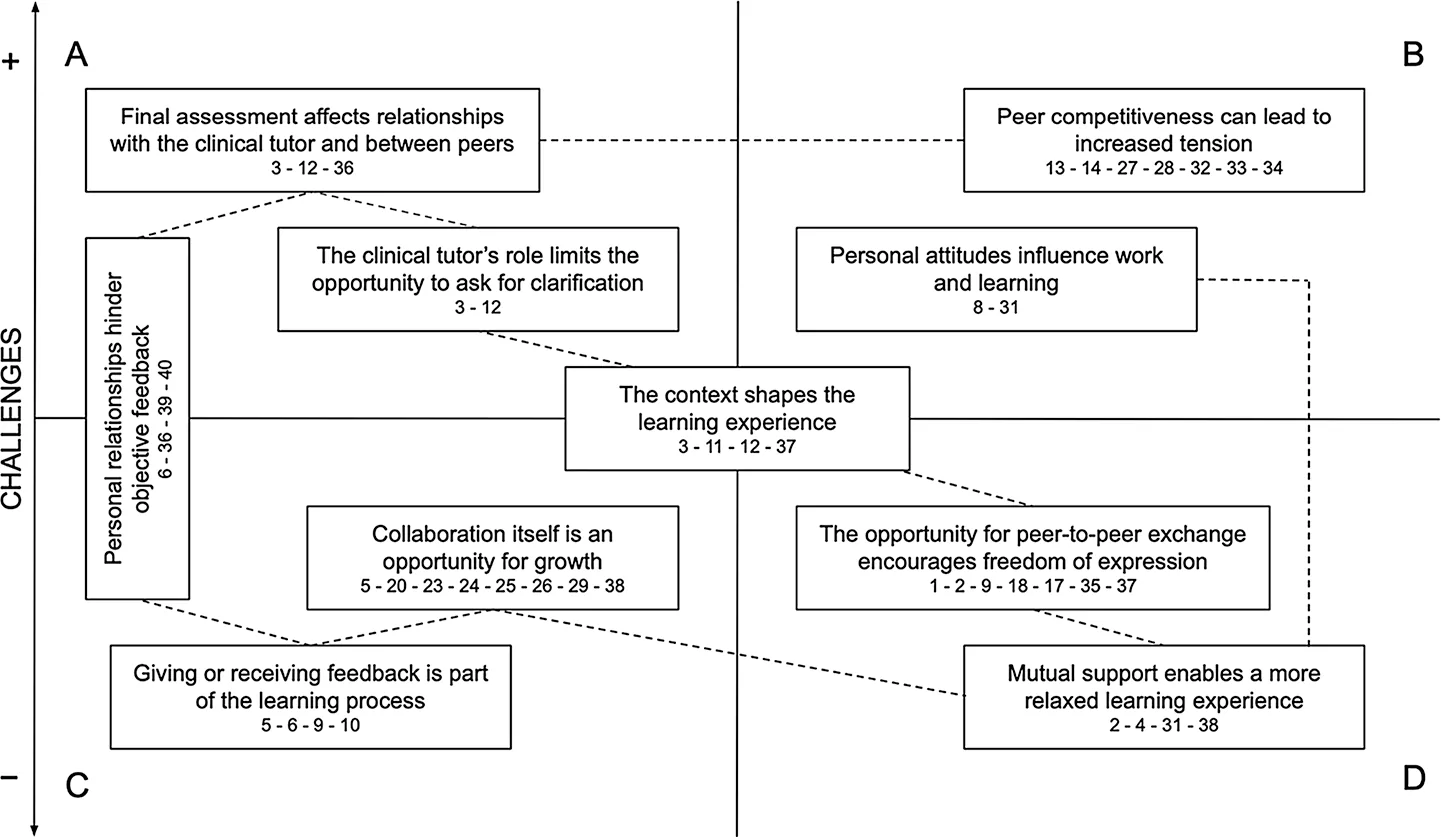
Graphical representation. (adapted from Byrne, 2022) of the themes (quadrants **A**, **B**, **C**, and **D**), the categories (boxes), and their interconnections (dashed lines), in relation to the increased or decreased perceived challenges during the peer-tutoring. Beyond analyzing only mentor and mentee quotations and considering all student experiences as those of peers due to their shared status as learners, the integrative synthesis identified the following key themes: (**A**) Indirect influence of the clinical tutor on peer relationship dynamics; (**B**) Presence of barriers to peer collaboration; (**C**) Promotion of personal growth; and (**D**) Reduction of difficulties associated with acting alone.

In addition to analyzing mentor and mentee quotations and considering all student experiences as those of peers due to their shared status as learners, the integrative synthesis identified the following key themes: (A) Indirect influence of the clinical tutor on peer relationship dynamics; (B) Presence of barriers to peer collaboration; (C) Promotion of personal growth; and (D) Reduction of difficulties associated with acting alone. These themes either increase or decrease the challenges perceived by students during the clinical experience, as shown in Fig. 3 (Numbered quotations in square brackets identify studies see Supplementary Information, Table S3).

### Theme A: indirect influence of the clinical tutor on peer relationship dynamics

Students’ narratives revealed tension in communication during clinical placements, particularly regarding how and with whom they felt comfortable expressing uncertainty or raising concerns. Participants frequently reported hesitations about asking questions they perceived as “silly” or basic when interacting with clinical tutors. These hesitations stemmed from fears of being judged or marked down, leading some students to withhold questions despite needing clarification.

Even just asking silly questions you don’t want to ask your supervisor because you think you might get marked down. It holds you back from asking some questions*.* (Sevenhuysen et al., 2015) [3]

In contrast, students reported feeling more comfortable discussing these uncertainties with peers, who were seen as more approachable and nonjudgmental. The peer relationship seemed to provide a psychologically safer space, allowing for more open dialogue, especially about clinical reasoning or gaps in knowledge.

It was helpful to discuss with your peers things you weren’t sure of or ‘stupid questions’ that you didn’t want to ask your educators. (Alpine et al., 2018) [12]

However, the data also revealed complexities in peer communication. While peers could serve as a supportive resource, students expressed discomfort addressing performance issues or providing critical feedback to one another, especially when a friendship had developed. The dual role of peers as both colleagues and friends created awkwardness around raising concerns, potentially limiting opportunities for constructive dialogue.

…it’s kind of hard to flag up any issues if we came across them because you’d kind of feel bad that they’re like a fellow student of yours… (Tailor et al., 2024) [36]

### Theme B: presence of barriers to peer collaboration

Students’ accounts reveal the subtle yet impactful dynamics of competition and comparison that can emerge in peer-assisted learning environments. Being placed alongside another student created situations in which individuals felt constrained in their ability to demonstrate competence, autonomy, or initiative. Some participants described feeling overshadowed or sidelined when peers dominated patient interactions, making it difficult to showcase their own skills and knowledge.

Hard to show how much you know when you’re constantly with another student. At times the other student tried to take over on my patients… (Alpine et al., 2018) [13]

The structure of shared placements also appeared to influence clinical decision-making. Students noted that once a peer initiated a particular approach to care, it became difficult to diverge from that path, limiting independent thinking and the expression of alternative clinical reasoning.

Because your peer has gone down one route then you kind of have to follow that… (Tailor et al., 2024) [32]

This dynamic sometimes led students to conform to peer choices instead of exploring their own. Additionally, students were often aware of and affected by implicit comparisons. The shared environment naturally led to informal benchmarking, with individuals evaluating their own performance against that of their peers. This ongoing comparison, whether related to feedback, patient interactions, or educator attention—could introduce pressure and self-doubt, and influence confidence and learning engagement.

You’re obviously kind of thinking like, how have I done in comparison to them… (Tailor et al., 2024) [33]

Participants noted that working closely with someone who approached tasks differently—whether in planning, execution, or interpretation—could create friction or inefficiency

My partner and [I] were quite different [in] the way we worked, the style of learning… (Sevenhuysen et al., 2015) [8]

These differences required negotiation and adaptability, which not all students found easy to manage, especially when coordination was lacking or mutual understanding was limited. Furthermore, students acknowledged moments when their peers made clinical choices or demonstrated behaviors they personally would not have adopted. This occasionally led to internal conflict or hesitation about how to respond, particularly when they were unsure whether to speak up or adapt silently to maintain harmony.

Sometimes your peer might do something that you wouldn’t maybe do… (Tailor et al., 2024) [31]

### Theme C: promotion of personal growth

Students described how working closely with peers fostered both professional and personal development. The shared learning experience encouraged them to grow together, strengthening not only their clinical skills but also their interpersonal connections

We grew as professionals together… made some new friends and built relationships. (Tailor et al., 2024) [38]

Feedback exchanges between peers emerged as a valuable and ongoing part of this growth. Students became comfortable giving and receiving constructive feedback, recognizing the unique effectiveness of peer-to-peer critique as it came from someone at a similar level of training

We got used to giving each other feedback and now we still do that even though we don’t have to… (Sevenhuysen et al., 2015) [6]

Having somebody at the same level as you offer feedback is very effective… (Alpine et al., 2018) [10]

This reciprocal feedback process contributed to reflective practice and mutual improvement.

### Theme D: reduction of difficulties associated with acting alone

Students reported that peer-tutoring facilitated deeper reflection and critical thinking. Learning closely with peers created opportunities to “bounce ideas off each other,” promoting active dialogue about clinical decisions and practice

I think I learnt more (in PAL). We helped each other to reflect… (Sevenhuysen et al., 2015) [2]

This collaborative engagement encouraged students to not only reflect on their own clinical skills but also evaluate the abilities of their peers, leading to a richer understanding of their own strengths and areas for improvement

2:1 placement allowed me to evaluate others’ abilities and reflect upon my own practice. (Alpine et al., 2018) [9]

Through this shared reflection, students felt their learning was enhanced compared to working alone.

Students reported that collaboration with a peer fostered a “safe space” to share thoughts, questions, and feedback without fear of judgment. This, in turn, helped them feel more at ease especially in the initial stages of their placements. The learning environment was identified as a central “binding” element across the identified themes (Fig. 3).

More comforting to have a student with you in the initial stages of your placement… (Alpine et al., 2018) [11]

There was less stress to it… I really enjoyed having, like, my peer with me… (Tailor et al., 2024) [37]

### Confidence in review findings

As reported in Tables S6–S10 (Supplementary Information), the GRADE-CERQual assessment showed that, among the four main themes identified, one was rated as having “Low confidence”: Theme A, “Indirect influence of the clinical tutor on peer relationship dynamics,” and three as “Moderate confidence”: Theme B, “Presence of barriers to peer collaboration”; Theme C, “Promotion of personal growth”; and Theme D, “Reduction of difficulties associated with acting alone.” Overall, the main concerns were related to methodological limitations, data relevance, and, in one case, adequacy. Common methodological issues included insufficient or missing information on sampling methods, data collection, researcher reflexivity, qualitative data analysis, and ethical considerations.

## Discussion

### Study main characteristics and their quality

To the best of our knowledge, this is the first meta-synthesis aimed at identifying, analysing, and synthesizing qualitative findings on the experiences of physiotherapy students participating in peer tutoring programs during their clinical internships. Only four studies have been identified, conducted in different countries, with just one (Paparella-Pitzel et al., 2021) specifically addressing the focus of this review. To generate insights relevant to educational practice, broad inclusion criteria were used. Although research in this area has received some attention over the past ten years, it remains limited, highlighting it as a priority for future investigation. In the nursing discipline, Jacobsen et al. (2022) found only ten qualitative studies, further suggesting that this is an overall priority in health care education. The quality of the studies retrieved in our review presented some methodological issues, which should be interpreted in the context of the complexity of such studies and the lack of appropriate guidelines for designing and conducting high-quality research. This suggests the need for recommendations to prevent any downgrade of the strength of the evidence produced.

### Meta summary and meta synthesis

In the meta-summary, the identified themes and subthemes were consistently present across nearly all four studies, indicating transversality and commonality in the potentialities and issues perceived by students during peer tutoring in clinical settings. The meta-synthesis highlighted that peer tutoring can reduce challenges by fostering personal and professional growth, enhancing collaborative skills, and alleviating the stress of working alone, thereby encouraging active participation in patient care and reflective learning. At the same time, it may introduce new challenges related to competitive dynamics, peer comparison, communication difficulties, and the indirect influence of clinical tutors on peer relationships, all of which can create tension, reduce autonomy, and compromise the overall quality of the learning experience. Therefore, the peer tutoring experience has both benefits and limitations, and its design and implementation should consider these emerging aspects to ensure full effectiveness for both mentees and mentors.

From the mentees’ perspective, findings align with an ethnographic study (Harder et al., 2013) showing that some students engaged in training activities based on high-fidelity simulation of clinical scenarios preferred active involvement over passive observation, as they believed the latter would negatively affect the quality of their training. Moreover, as in the nursing field (Jacobsen et al., 2022), mentees believed peer interactions were more effective than those with clinical tutors, as peer interaction considerably reduced anxiety. The deconstruction of the asymmetrical teacher-student relationship established different foundations for learning: mentors generally perceived their role as supporting and guiding students within a safe learning environment, facilitating moments of exchange and discussion (Christensen et al., 2023).

Similarly, consistent with our meta-synthesis findings, a mixed-methods study in the field of peer-led simulation activities (Granger et al., 2024) found that second-year students serving as “peer-teachers” (mentors) reported personal benefits such as increased confidence, improved communication, greater empathy, enhanced feedback skills, and the consolidation and self-reflection of their clinical and theoretical knowledge. These outcomes contributed to the development of their professional identity and support the idea that teaching is considered the most effective way to learn (e.g., Ytreberg & Aars, 2014). According to Jacobsen et al. (2022), in their meta-synthesis on nursing education, being a mentor led students to engage in deep reflection on their competencies, expand their knowledge to teach peers, and develop a strong sense of responsibility.

Knowing how to provide and receive feedback requires critically assessing one’s own and others’ actions while avoiding biased positions influenced by interpersonal relationships. “Peer assessment” (Li et al., 2019; Stenberg et al., 2021) has attracted considerable interest in higher education for its educational value and its ability to offer students opportunities to develop important transversal skills. Although it has positive effects (Maas et al., 2014), in clinical internships where a clinical tutor is responsible for assessing student competencies, it is important to consider that giving feedback on a peer, especially to the clinical tutor, may sometimes become a tool for achieving one’s own ultimate goal—obtaining a good evaluation (McGarr & Clifford, 2013).

Finally, regarding challenges experienced during clinical internships, the literature shows that peer tutoring strategies can reduce anxiety levels (e.g., Kachaturoff et al., 2020; Jacobsen et al., 2022). However, our results suggest that certain situations may have the opposite effect, such as when students are asked to provide potentially “uncomfortable” feedback about a peer to the clinical tutor, fully aware of the tutor’s role as evaluator and the impact of evaluation on their academic trajectory. Similarly, harmful competitiveness among students, driven by the pursuit of a positive evaluation from the clinical tutor, could negatively affect stress and anxiety levels. In the first case, stress may arise from the intention not to put a peer or friend at a disadvantage, while in the second case, the primary cause may be the need for an individual to stand out over others. Regarding competitiveness, potential incompatibilities among students, and relationships with clinical tutors, can trigger competition. They are aware of being evaluated during clinical internships and, in some cases, may suspect that the clinical tutor favors one student over another. However, as found in our review, peer tutoring may also mitigate this competitiveness, as students in both mentee and mentor roles recognized the development of cooperative skills.

Difficulties in ensuring adequate clinical internships have long been recognized (e.g., McMahon et al., 2014), with the shortage of clinical tutors being a major challenge, both in terms of absolute numbers and the limited tradition or willingness to assume the role of clinical tutor, especially in primary care settings. According to the findings, although more research is needed, peer tutoring can be considered a valuable experience, balancing its pros and cons. However, the student mentor should be trained and supervised by the clinical tutor regarding their mentoring role, which requires additional competencies and resources to be allocated (Christensen et al., 2023; Alpine et al., 2018). Alternative educational models should be considered to address limitations in clinical internship opportunities (e.g., O’Connor et al., 2012), such as simulations and training activities without direct patient care (Granger et al., 2024; Christensen et al., 2023).

### Strengths and limitations

The strength of this meta-synthesis lies in its comprehensive search strategy across the most relevant databases. Although the number of available studies on the topic was limited, the findings were consistent and homogeneous. Conducting the various phases of the research independently before reaching consensus helped ensure the credibility of the results and increased confidence in the interpretations drawn from the data.

However, this review has several limitations. First, the search string did not include the terms “mentee” and “mentor” and relied mainly on widely used keywords; therefore, despite the rigorous approach, some relevant studies may have been missed. Second, studies focused on clinical internship models with different tutor-student ratios were excluded unless they explicitly involved peer mentoring. Third, we included all retrieved studies regardless of methodological quality. While some scholars (e.g., Estabrooks et al., 1994) argue that a pragmatic and inclusive approach to quality assessment may be a limitation, others (e.g., Herber & Barroso, 2020) criticize the exclusion of studies based on technical or descriptive shortcomings that do not compromise the credibility of their findings (Garside, 2014).

## Conclusions

To date, only a few studies have examined the experiences of physiotherapy students participating in peer tutoring-based clinical internships. The four identified studies, conducted in different countries and characterized by some methodological limitations, provided evidence of overall moderate quality. Clinical internships that include peer tutoring appear to have both positive and negative effects, either alleviating or intensifying the challenges physiotherapy students encounter in complex healthcare settings.

Peer tutoring can benefit both mentors and mentees. Students supported by peers during clinical placements—whether as mentors, mentees, or equal peers—may find it easier to learn and develop core competencies essential for professional practice. Peer collaboration can enhance reflective thinking, promote mutual support, and foster a sense of safety during the learning process. However, the available studies also identify potential negative dynamics. Peer relationships may be affected by competitiveness, interpersonal tensions, or differences in learning styles and work rhythms, which can increase stress or hinder collaboration. Additionally, the dual role of the clinical tutor as both supervisor and evaluator may limit the openness of peer feedback and contribute to student anxiety, especially when feedback about peers could influence final evaluations.

### Implications for clinical practice

Peer tutoring-based clinical internships should be carefully designed, prepared, and implemented, supported by structured supervision and specific mentor training to avoid unintended negative consequences and ensure a constructive learning environment. Peer tutoring programs should be implemented within a structured and well-supervised framework to provide a safe and effective learning experience. Specific training for mentors is essential, particularly in developing communication skills, delivering feedback, and managing relationships. Clinical tutors should be aware of the potential impact of their dual roles as supervisors and evaluators on peer dynamics and adopt strategies that foster trust and collaboration. Continuous monitoring of group interactions is necessary to prevent harmful competitiveness and promote a shared, supportive learning environment.

### Implications for future research

Further qualitative research with a rigorous methodological approach is needed to gather evidence on physiotherapy students’ experiences with peer tutoring. Including diverse settings is encouraged to generate evidence across varied learning contexts, such as high-acuity and home-based care environments. The effectiveness of strategies to prepare students as mentors should be assessed, and the experiences of clinical tutors in these models should be documented to provide evidence regarding all participants: students, mentees, mentors, and physiotherapists serving as clinical tutors. Additionally, the long-term effects of peer tutoring on professional identity development and overall training quality require investigation.

## Supplementary Information

Below is the link to the electronic supplementary material.

Supplementary Material 1

## Data Availability

No datasets were generated or analysed during the current study.
