# Supplementary material for: Peer tutoring during clinical internships as experienced by physiotherapy students: a meta-synthesis of qualitative studies
Source: Adv Health Sci Educ Theory Pract. 2025 Nov 7;31(4):1303–26. doi: 10.1007/s10459-025-10488-7 (PMC13553645; doi:10.1007/s10459-025-10488-7)
Supplement: Supplementary file 1 [file 10459_2025_10488_MOESM1_ESM.docx]

**The Experience of Peer Tutoring During Clinical Internships of Physiotherapy Students: A Meta-Synthesis of Qualitative Studies**

**Simone Salvitti^a,c^, Isabel Colado Gimeno^b^, Alvisa Palese^c^, Irene Mansutti^c^, Mauro Di Bari^d,e^**

^a^ Unit of Cardiorespiratory Physiotherapy, Azienda Sanitaria Universitaria Friuli Centrale (ASUFC), Udine, Italy

^b^ Unit of Pediatric Physiotherapy, Azienda Sanitaria Universitaria Friuli Centrale (ASUFC), Udine, Italy

^c^ Department of Medicine, University of Udine, Italy

^d^ Department of Experimental and Clinical Medicine, University of Florence, Italy

^e^ Unit of Geriatrics, Department of Medicine and Geriatrics, Azienda Ospedaliero-Universitaria Careggi, Florence, Italy

**Corresponding author:** Simone Salvitti (ORCID: 0000-0002-9732-6255); Azienda Sanitaria Universitaria Friuli Centrale (ASUFC), Department of Rehabilitation Health Professions, General Rehabilitation Platform, Cardiorespiratory Unit – 330 Pozzuolo Street, 33100 Udine (Italy). simonesalvitti@gmail.com

**SUPPLEMENTARY INFORMATION**

**Figure S1**. Overview graph comparing the number of physiotherapists employed within Local Health Authorities (ASLs) and public or equivalent inpatient institutions, with the number of students enrolled in undergraduate Physiotherapy programs at public and private universities in Italy. The number of physiotherapists was estimated as 60% (based on Ministry of Health reports for the years 2011, 2018, 2019, and 2020) of the total rehabilitation workforce calculated for each calendar year. The number of students was calculated as the sum of the positions announced by the Ministry of Education for the corresponding three-year period (e.g., number of students in 2024 = students enrolled in 2022 [3rd year] + 2023 [2nd year] + 2024 [1st year]).

**References:**

Ministry of Health reports for the years 2011, 2018, 2019, and 2020

- 2011: <https://www.salute.gov.it/imgs/C_17_pubblicazioni_2191_allegato.pdf>
- 2018: <https://www.salute.gov.it/imgs/C_17_pubblicazioni_3011_allegato.pdf>
- 2019: <https://www.salute.gov.it/imgs/C_17_pubblicazioni_3164_allegato.pdf>
- 2020: <https://www.salute.gov.it/imgs/C_17_pubblicazioni_3273_allegato.pdf>

Ministry of Education

- <https://www.mim.gov.it/web/guest/normativa>

**Figure S2**. Minimum number of clinical tutors required to meet training needs, according to the Guidelines for the definition of agreements pursuant to Article 6, paragraph 3, of Legislative Decree 502/92 and subsequent amendments, for the training of healthcare professionals as outlined in Law 251/2000, and Ministerial Decree No. 234 of September 24, 1997, which defines a maximum clinical tutor-to-student ratio of 1:2. Values shown for each academic year represent half the ratio between the total number of internship hours to be delivered (calculated as the product of the number of students per year and 500 internship hours, based on an average of 1500 hours over the three-year program) and the number of annual working hours available per tutor, net of holidays, leave, and sick days (estimated at 1500 hours/year, corresponding to approximately 80% of the total annual hours for a full-time contract of 36 hours/week over 52 weeks).

**References:**

- Legislative Decree 502/92
  - <https://www.normattiva.it/uri-res/N2Ls?urn:nir:stato:decreto.legislativo:1992-12-30;502>
- Law 251/2000
  - <https://www.gazzettaufficiale.it/eli/id/2000/09/06/000G0299/sg>
  - <https://www.quotidianosanita.it/allegati/allegato1658866418.pdf>
- Ministerial Decree 234/97
  - <https://www.gazzettaufficiale.it/atto/serie_generale/caricaDettaglioAtto/originario?atto.dataPubblicazioneGazzetta=1997-10-07&atto.codiceRedazionale=097A7759&elenco30giorni=false>

**Figure S3**. Search string for checking existence of meta-synthesis

**Figure S4**. Search string for querying databases

| **Critical appraisal** | **CASP** | **1** | **2** | **3** | **4** | **5** | **6** | **7** | **8** | **9** | **10** | **Total** |
| --- | --- | --- | --- | --- | --- | --- | --- | --- | --- | --- | --- | --- |
| Low | Tailor et al., 2024 | Y | Y | Y | N | C | N | C | C | Y | Y | 5 |
| Low | Paparella-Pitzel et al., 2021 | Y | Y | Y | N | Y | N | C | C | N | Y | 5 |
| Moderate | Sevenhuysen et al., 2015 | Y | Y | Y | N | C | N | Y | N | Y | Y | 6 |
| Very Low | Alpine at al., 2018 | C | N | N | N | N | N | N | N | N | Y | 1 |

Legend: Y: Yes; C: Can’t tell; N: No

**Table S1**. Overall assessment of the reporting quality of the included studies using the Critical Appraisal Skills Programme (CASP) checklist.

| **n** | **QUESTIONS** | **Y** | **C** | **N** |
| --- | --- | --- | --- | --- |
| 1 | Was there a clear statement of the aims of the research? | 3 | 1 | 0 |
| 2 | Is a qualitative methodology appropriate? | 3 | 0 | 1 |
| 3 | Was the research design appropriate to address the aims of the research? | 3 | 0 | 1 |
| 4 | Was the recruitment strategy appropriate to the aims of the research? | 0 | 0 | 4 |
| 5 | Was the data collected in a way that addressed the research issue? | 1 | 2 | 1 |
| 6 | Has the relationship between researcher and participants been adequately considered? | 0 | 0 | 4 |
| 7 | Have ethical issues been taken into consideration? | 1 | 2 | 1 |
| 8 | Was the data analysis sufficiently rigorous? | 0 | 2 | 2 |
| 9 | Is there a clear statement of findings? | 2 | 0 | 2 |
| 10 | How valuable is the research? | 4 | 0 | 0 |

Legend: Y: Yes; C: Can’t tell; N: No

**Table S2**. Overall assessment of individual items of the included studies using the Critical Appraisal Skills Programme (CASP) checklist.

**Sevenhuysen et al., 2015**

1. “Instead of just being thrown in the deep end, to do a subjective [history taking] on your own, complete an assessment on your own, it was good to have that person there to bounce ideas off. We could write out a plan together and we followed through together. Just having the confidence, reliance on someone else, made it easier”

2. “I think I learnt more (in PAL). We helped each other to reflect. You could talk about what you did and how you could do it differently. We would sit down and debrief with each other and go ‘how can we be different tomorrow?”

3. “Even just asking silly questions you don’t want to ask your supervisor because you think you might get marked down. It holds you back from asking some questions”

4. “It’s just being able to bounce things off each other. Our supervisor mentioned that she likes that we could work together, and we felt good about being able to rely on each other”

5. “I really appreciated when my peer gave me feedback. It’s just a different perspective from the supervisor as well”

6. “We got used to giving each other feedback and now we still do that even though we don’t have to… So I guess sometimes you might think you don’t want to tell them, offend them, but because we had to in the beginning now we just keep giving each other feedback”

7. “It [feedback from the clinical educator]... was more in depth and... more relevant. It might have been that I respect the opinion of the [educator]. Not that I don’t of my peer, but you respect your [educator] a lot more because they have the experience and really know what they’re talking about”

8. “My partner and [I] were quite different [in] the way we worked, the style of learning. It was hard to coordinate that because I would learn a different way to how he would. Working together wasn’t so easy”

**Alpine at al., 2018**

9. “2:1 placement allowed me to evaluate others abilities as a physiotherapist and have more time to reflect upon my own practice. The opportunity was given to compare others strengths and weaknesses against my own and adapt my practice accordingly”

10. “Having somebody at the same level as you offer feedback is very effective. Similarly, watching another student carry out an assessment/treatment and having to offer feedback is a learning experience for yourself, as you offer critical feedback on mistakes which you may have made yourself”

11. “More comforting to have a student with you in the initial stages of your placement. Less nervous than if I was on my own”

12. “It was helpful to discuss with your peers things you weren’t sure of or “stupid questions” that you didn’t want to ask your educators”

13. “Hard to show how much you know when you’re constantly with another student. At times the other student tried to take over on my patients. It can provide an opportunity for one student to try to show up the other student or make them look bad”

14. “It allowed the weaker student to learn and progress more while the stronger student took a more laid back and un-stimulating position”

**Paparella-Pitzel et al., 2021**

15. “Their (senior peer) assessment skills surprised me in the beginning. They were good”

16. “Working with the 2nd year helped when I was a 1st year, but sometimes I felt I was of no use because the student PTs to patient ratio”

17. “Working in pairs of 2 students per 1 patient helped me learn better”

18. “Working with another student was beneficial because you can bounce ideas off of each other which was helpful”

19. “They (senior peer) seemed to keep the treatment based around the patient and it seemed like the right move”

20. “There were some instances in which a student did something and I had no idea what it was for or why until they explained it to me”

21. “First visit, I was host and got to build a rapport with the patients in the front which was nice. Second visit, I was inside, and it was fun to watch patients working hard and getting better through movement as well as working with peers to come up with the best program possible”

22. “1st year I felt useless and was never asked to do anything, 2nd year I tried to incorporate the first to teach them and treat with me so they could have a better experience”

23. “Experience of working with peers to come up with the best plan of care for the patient”

24. “I feel like I worked collaboratively to develop interventions”

25. “I liked being able to co‐treat to gain experience”

26. “When I worked with a 2nd‐year student, it flowed better versus me treating by myself because we made sure to be comprehensive”

27. “One guy (1st year) got too cocky and started interrupting me with wrong information”

28. “Sometimes they (1st year students) would try to correct us too much. They were at times a bit too confident”

29. “(The biggest benefit to participating in clinic was the) experience of working with peers to come up with the best plan of care for the patient”

30. “The second visit was more interesting: the 2nd‐year student allowed me to be more hands‐on with the patient”

**Tailor et al., 2024**

31. “Sometimes your peer might do something that you wouldn’t maybe do but I think it’s about getting that balance and also maybe not being selfish with it, knowing that other people need their time to do it”

32. “Because your peer has gone down one route then you kind of have to follow that... it is all about the individual and sometimes the peer work kind of masks what you could do yourself potentially”

33. “You’re obviously kind of thinking like, how have I done in comparison to them.(...) suppose you always have that kind of like wonder, have they marked me better than them?”

34. “I feel like the other person didn’t get as much time with the band 7 as what I did... I got to see kind of more interesting patients and got to go to different wards and stuff whereas she didn’t get to really have that experience”

35. “When you’re on your own you’re forced to talk to like other people and like make those relationships. But when it was me and my friend we’d talk together and then in a group”

36. “...it’s kind of hard to flag up any issues if we came across them because you’d kind of feel bad that they’re like a fellow student of yours so like. It’s almost because like you’re working with a friend, that if they did something wrong or you wanted to give them feedback about something, or like you needed to mention to your educator something like “I think this happened earlier and I don’t think it was right” or something, it’s quite awkward to do because obviously like, they’re like one of your friends so you don’t like mentioning it”

37. “There was less stress to it that we could freely talk about our clinical reasoning without worrying about being completely wrong (...) I really enjoyed having, like, my peer with me as opposed to my educator, it felt like you could be that little bit more confident”

38. “At first, we didn’t really know each other at all and then by the end of it we were really good mates and (…) grew as professionals together which was quite nice as well. So not only did we like, push each other, and help each other in the placement we also (...), made some new friends and built relationships”

39. “I suppose depending on the relationship you’ve got (...) I’d feel much better to bring up something positive feedback, like “you did really well”

40. “It took me (...) longer to understand how they worked and like what they were like as a person (...) it’s also quite good because if it was one of your really close friends, if you ended up clashing or disagreeing it would kind of make it quite awkward

**Table S3.** Quotations extracted from studies

| **Themes** | **Subthemes** | **Quotations** |
| --- | --- | --- |
| Reduction of difficulties associated with acting alone | Opportunity for peer discussion | “Instead of just being thrown in the deep end, to do a subjective [history taking] on your own, complete an assessment on your own, it was good to have that person there to bounce ideas off. We could write out a plan together and we followed through together. Just having the confidence, reliance on someone else, made it easier” |
|  |  | “I think I learnt more [in PAL]. We helped each other to reflect. You could talk about what you did and how you could do it differently. We would sit down and debrief with each other and go ‘how can we be different tomorrow?” |
|  |  | “2:1 placement allowed me to evaluate others abilities as a physiotherapist and have more time to reflect upon my own practice. The opportunity was given to compare others strengths and weaknesses against my own and adapt my practice accordingly” |
|  |  | “Working with another student was beneficial because you can bounce ideas off of each other which was helpful” |
|  |  | “Working in pairs of 2 students per 1 patient helped me learn better” |
|  |  | “When you’re on your own you’re forced to talk to like other people and like make those relationships. But when it was me and my friend we’d talk together and then in a group” |
|  |  | “There was less stress to it that we could freely talk about our clinical reasoning without worrying about being completely wrong (...) I really enjoyed having, like, my peer with me as opposed to my educator, it felt like you could be that little bit more confident” |
|  | Mutual support | “It’s just being able to bounce things off each other. Our supervisor mentioned that she likes that we could work together, and we felt good about being able to rely on each other” |
|  |  | “At first, we didn’t really know each other at all and then by the end of it we were really good mates and (…) grew as professionals together which was quite nice as well. So not only did we like, push each other, and help each other in the placement we also (...), made some new friends and built relationships” |
|  | Safe environment | “Even just asking silly questions you don’t want to ask your supervisor because you think you might get marked down. It holds you back from asking some questions” |
|  |  | “More comforting to have a student with you in the initial stages of your placement. Less nervous than if I was on my own” |
|  |  | “It was helpful to discuss with your peers things you weren’t sure of or “stupid questions” that you didn’t want to ask your educators” |
|  |  | “There was less stress to it that we could freely talk about our clinical reasoning without worrying about being completely wrong (...) I really enjoyed having, like, my peer with me as opposed to my educator, it felt like you could be that little bit more confident” |
| Personal growth | Enhancing feedback-giving skills | “I really appreciated when my peer gave me feedback. It’s just a different perspective from the supervisor as well” |
|  |  | “We got used to giving each other feedback and now we still do that even though we don’t have to… So I guess sometimes you might think you don’t want to tell them, offend them, but because we had to in the beginning now we just keep giving each other feedback” |
|  |  | “Having somebody at the same level as you offer feedback is very effective. Similarly, watching another student carry out an assessment/treatment and having to offer feedback is a learning experience for yourself, as you offer critical feedback on mistakes which you may have made yourself” |
|  |  | “Sometimes your peer might do something that you wouldn’t maybe do but I think it’s about getting that balance and also maybe not being selfish with it, knowing that other people need their time to do it” |
|  | Developing collaborative abilities | “I really appreciated when my peer gave me feedback. It’s just a different perspective from the supervisor as well” |
|  |  | “I feel like I worked collaboratively to develop interventions” |
|  |  | “I liked being able to co‐treat to gain experience” |
|  |  | “Experience of working with peers to come up with the best plan of care for the patient” |
|  |  | “(The biggest benefit to participating in clinic was the) experience of working with peers to come up with the best plan of care for the patient” |
|  |  | “When I worked with a 2nd‐year student, it flowed better versus me treating by myself because we made sure to be comprehensive” |
|  |  | “There were some instances in which a student did something and I had no idea what it was for or why until they explained it to me” |
|  |  | “At first, we didn’t really know each other at all and then by the end of it we were really good mates and (…) grew as professionals together which was quite nice as well. So not only did we like, push each other, and help each other in the placement we also (...), made some new friends and built relationships” |
| Collaboration difficulties | Inappropriate competitiveness | “Hard to show how much you know when you’re constantly with another student. At times the other student tried to take over on my patients. It can provide an opportunity for one student to try to show up the other student or make them look bad” |
|  |  | “It allowed the weaker student to learn and progress more while the stronger student took a more laid back and un-stimulating position” |
|  |  | “One guy (1st year) got too cocky and started interrupting me with wrong information” |
|  |  | “Sometimes they (1st year students) would try to correct us too much. They were at times a bit too confident” |
|  |  | “Because your peer has gone down one route then you kind of have to follow that... it is all about the individual and sometimes the peer work kind of masks what you could do yourself potentially” |
|  |  | “You’re obviously kind of thinking like, how have I done in comparison to them.(...) suppose you always have that kind of like wonder, have they marked me better than them?” |
|  |  | “I feel like the other person didn’t get as much time with the band 7 as what I did... I got to see kind of more interesting patients and got to go to different wards and stuff whereas she didn’t get to really have that experience” |
|  | Differences in learning and working styles | “My partner and [I] were quite different [in] the way we worked, the style of learning. It was hard to coordinate that because I would learn a different way to how he would. Working together wasn’t so easy” |
|  |  | “Sometimes your peer might do something that you wouldn’t maybe do but I think it’s about getting that balance and also maybe not being selfish with it, knowing that other people need their time to do it” |
|  | Perceived lack of usefulness | “Working with the 2nd year helped when I was a 1st year, but sometimes I felt I was of no use because the student PTs to patient ratio” |
|  |  | “1st year I felt useless and was never asked to do anything, 2nd year I tried to incorporate the first to teach them and treat with me so they could have a better experience” |
|  |  | “The second visit was more interesting: the 2nd‐year student allowed me to be more hands‐on with the patient” |
| Relationship with the clinical tutor | Fear of asking questions perceived as silly | “Even just asking silly questions you don’t want to ask your supervisor because you think you might get marked down. It holds you back from asking some questions” |
|  |  | “It was helpful to discuss with your peers things you weren’t sure of or “stupid questions” that you didn’t want to ask your educators” |
|  | Subjective or biased feedback | “...it’s kind of hard to flag up any issues if we came across them because you’d kind of feel bad that they’re like a fellow student of yours so like. It’s almost because like you’re working with a friend, that if they did something wrong or you wanted to give them feedback about something, or like you needed to mention to your educator something like “I think this happened earlier and I don’t think it was right” or something, it’s quite awkward to do because obviously like, they’re like one of your friends so you don’t like mentioning it” |
|  |  | “I suppose depending on the relationship you’ve got (...) I’d feel much better to bring up something positive feedback, like “you did really well” |

Legend: in orange: quotations from “mentees”; in yellow: quotations from “mentors”

**Table S4.** Meta-summary table

|  | **Quotations** | **Conceptualizations** | **Subthemes** | **Themes** |
| --- | --- | --- | --- | --- |
| **Mentee** | “Working with the 2^nd^ year helped when I was a 1^st^ year, but sometimes I felt I was of no use because the student PTs to patient ratio” | Becoming aware of the need to prioritize hands-on clinical practice | Perceived lack of usefulness  The mentee wants to be hands-on and not limited to observation | **Actively participating in patient care** |
|  | “1^st^ year I felt useless and was never asked to do anything, 2^nd^ year I tried to incorporate the first to teach them and treat with me so they could have a better experience” |  |  |  |
|  | “The second visit was more interesting: the 2^nd^‐year student allowed me to be more hands‐on with the patient” |  |  |  |
|  | “Experience of working with peers to come up with the best plan of care for the patient” | Recognizing collaboration with the mentor as a strategy to improve the quality and effectiveness of care | Development of collaborative skills  Collaborating with the mentor to achieve patient-related outcomes  Mentor as a role model  Engaging with the mentee as a part of the learning process |  |
|  | “(The biggest benefit to participating in clinic was the) experience of working with peers to come up with the best plan of care for the patient” |  |  |  |
|  | “When I worked with a 2^nd^‐year student, it flowed better versus me treating by myself because we made sure to be comprehensive” |  |  |  |
|  | “...it was fun to watch patients working hard and getting better through movement as well as working with peers to come up with the best program possible” | Identifying the mentor as the main reference figure for learning during clinical placement |  |  |
|  | “Their (senior peer) assessment skills surprised me in the beginning. They were good” |  |  |  |
|  | “They (senior peer) seemed to keep the treatment based around the patient and it seemed like the right move” |  |  |  |
| **Mentor** | “Working with another student was beneficial because you can bounce ideas off of each other which was helpful” | Recognizing the opportunity to engage with the mentee as beneficial for learning |  | **Developing a path of personal and professional growth** |
|  | “Working in pairs of 2 students per 1 patient helped me learn better” |  |  |  |
|  | “I feel like I worked collaboratively to develop interventions” | Viewing collaboration with the mentee as a way to enhance one's own skills | Opportunity for comparison  Collaboration with the mentee as a way to refine technique |  |
|  | “I liked being able to co‐treat to gain experience” |  |  |  |
|  | “One guy (1^st^ year) got too cocky and started interrupting me with wrong information” | Acknowledging that some mentees may feel the need to demonstrate their own abilities and competences | Inappropriate competitiveness  Mentees sometimes take over the mentor’s role |  |
|  | “Sometimes they (1^st^ year students) would try to correct us too much. They were at times a bit too confident” |  |  |  |

**Table S5**. Integrative synthesis of the quotations of mentors and mentee (Paparella-Pitzel et al., 2021)

| **Summary of Findings** | **Articles Contributing to the Findings** | **GRADE-CERQual Confidence Rating** | **GRADE-CERQual Evaluation Description** |
| --- | --- | --- | --- |
| **Theme A**: Indirect influence of the clinical tutor on peer relationship dynamics | Sevenhuysen et al., 2015; Alpine at al., 2018; Tailor et al., 2024 | Low confidence | Downgraded due to serious concerns regarding methodological limitations and minor concerns regarding relevance and adequacy |
| **Theme B**: Presence of barriers to peer collaboration | Sevenhuysen et al., 2015; Alpine at al., 2018; Paparella-Pitzel et al., 2021; Tailor et al., 2024 | Moderate confidence | Downgraded due to serious concerns regarding methodological limitations and minor concerns regarding relevance |
| **Theme C**: Promotion of personal growth | Sevenhuysen et al., 2015; Alpine at al., 2018; Paparella-Pitzel et al., 2021; Tailor et al., 2024 | Moderate confidence | Downgraded due to serious concerns regarding methodological limitations and minor concerns regarding relevance |
| **Theme D**: Reduction of difficulties associated with acting alone | Sevenhuysen et al., 2015; Alpine at al., 2018; Paparella-Pitzel et al., 2021; Tailor et al., 2024 | Moderate confidence | Downgraded due to serious concerns regarding methodological limitations and minor concerns regarding relevance |

Legend: GRADE-CERQUAL: Grading of Recommendations Assessment, Development and Evaluation - Confidence in the Evidence from Reviews of Qualitative research

**Table S6**. Summary of Qualitative Findings (GRADE-CERQual)

| **Theme A**: Indirect influence of the clinical tutor on peer relationship dynamics | |
| --- | --- |
| Methodological Limitations | Serious concerns – studies showed several methodological limitations such as incomplete or entirely missing information regarding sampling methods, data collection processes, researcher reflexivity, and qualitative data analysis |
| Coherence | No or very minor concerns – a plausible alternative interpretation to those of the original authors may exist, but no contradictions were found in the extracted and analyzed data |
| Adequacy | Minor concerns – two studies provided sufficiently detailed information, while one study presented more general and superficial data |
| Relevance | Minor concerns – although conducted in different settings, the studies collected the experiences of physiotherapy students in high-income countries |
| Overall GRADE-CERQual confidence and justification | |
| Low confidence | Downgraded due to serious concerns about methodological limitations and minor concerns regarding relevance and adequacy |

Legend: GRADE-CERQUAL: Grading of Recommendations Assessment, Development and Evaluation - Confidence in the Evidence from Reviews of Qualitative research

**Table S7**. Theme A Qualitative Findings (GRADE-CERQual)

| **Theme B**: Presence of barriers to peer collaboration | |
| --- | --- |
| Methodological Limitations | Serious concerns – several methodological limitations including incomplete or missing details on sampling, data collection, researcher reflexivity, and data analysis |
| Coherence | No or very minor concerns – no inconsistencies found; the findings are plausibly interpreted |
| Adequacy | No or very minor concerns – all studies provided sufficiently detailed information |
| Relevance | Minor concerns – although conducted in various settings, all studies referred to physiotherapy students in high-income countries |
| Overall GRADE-CERQual confidence and justification | |
| Moderate confidence | Downgraded due to serious methodological concerns and minor concerns regarding relevance |

Legend: GRADE-CERQUAL: Grading of Recommendations Assessment, Development and Evaluation - Confidence in the Evidence from Reviews of Qualitative research

**Table S8**. Theme B - Qualitative Findings (GRADE-CERQual)

| **Theme C**: Promotion of personal growth | |
| --- | --- |
| Methodological Limitations | Serious concerns – major limitations regarding sampling, data collection, researcher reflexivity, and analysis methods |
| Coherence | No or very minor concerns – findings are consistent and plausible |
| Adequacy | No or very minor concerns – all studies provided adequately detailed information |
| Relevance | Minor concerns – settings differed, but the target population remained consistent (physiotherapy students in high-income countries) |
| Overall GRADE-CERQual confidence and justification | |
| Moderate confidence | Downgraded due to serious concerns regarding methodological limitations and minor concerns about relevance |

Legend: GRADE-CERQUAL: Grading of Recommendations Assessment, Development and Evaluation - Confidence in the Evidence from Reviews of Qualitative research

**Table S9**. Theme C - Qualitative Findings (GRADE-CERQual)

| **Theme D**: Reduction of difficulties associated with acting alone | |
| --- | --- |
| Methodological Limitations | Serious concerns – consistent methodological issues across studies regarding sampling, data collection, reflexivity, and analysis |
| Coherence | No or very minor concerns – no conflicting findings were observed |
| Adequacy | No or very minor concerns – information provided was sufficiently detailed in all studies |
| Relevance | Minor concerns – despite variability in setting, the population studied remained within the same professional and socioeconomic context |
| Overall GRADE-CERQual confidence and justification | |
| Moderate confidence | Downgraded due to serious concerns regarding methodological limitations and minor concerns about relevance |

Legend: GRADE-CERQUAL: Grading of Recommendations Assessment, Development and Evaluation - Confidence in the Evidence from Reviews of Qualitative research

**Table S10**. Theme D - Qualitative Findings (GRADE-CERQual)
